# Quantifying growing versus non-growing ovarian follicles in the mouse

**DOI:** 10.1186/s13048-016-0296-x

**Published:** 2017-01-13

**Authors:** Bahar Uslu, Carola Conca Dioguardi, Monique Haynes, De-Qiang Miao, Meltem Kurus, Gloria Hoffman, Joshua Johnson

**Affiliations:** 1 Department of Obstetrics, Gynecology, and Reproductive Sciences, Yale School of Medicine, 333 Cedar Street, New Haven, Connecticut USA; 2 Current Address: Center for Reproductive Biology, Washington State University, PO Box 647521, Pullman, 99164 Washington USA; 3 Department of Histology and Embryology, Izmir Katip Celebi University School of Medicine, Izmir, Turkey; 4 Department of Biology, Morgan State University, 1700 East Cold Spring Lane, Baltimore, 21251 Maryland USA; 5 Department of Obstetrics and Gynecology, University of Colorado-Denver, Building RC2, Room P15 3103, Aurora, 80045 Colorado USA

**Keywords:** Atresia, Follicle, Fragile X, Histomorphometric evaluation, Oocyte, Ovarian reserve, Ovary, Premutation

## Abstract

**Background:**

A standard histomorphometric approach has been used for nearly 40 years that identifies atretic (e.g., dying) follicles by counting the number of pyknotic granulosa cells (GC) in the largest follicle cross-section. This method holds that if one pyknotic granulosa nucleus is seen in the largest cross section of a primary follicle, or three pyknotic cells are found in a larger follicle, it should be categorized as atretic. Many studies have used these criteria to estimate the fraction of atretic follicles that result from genetic manipulation or environmental insult. During an analysis of follicle development in a mouse model of Fragile X premutation, we asked whether these ‘historical’ criteria could correctly identify follicles that were not growing (and could thus confirmed to be dying).

**Methods:**

Reasoning that the fraction of mitotic GC reveals whether the GC population was increasing at the time of sample fixation, we compared the number of pyknotic nuclei to the number of mitotic figures in follicles within a set of age-matched ovaries.

**Results:**

We found that, by itself, pyknotic nuclei quantification resulted in high numbers of false positives (improperly categorized as atretic) and false negatives (improperly categorized intact). For preantral follicles, scoring mitotic and pyknotic GC nuclei allowed rapid, accurate identification of non-growing follicles with 98% accuracy. This method most often required the evaluation of one follicle section, and at most two serial follicle sections to correctly categorize follicle status. For antral follicles, we show that a rapid evaluation of follicle shape reveals which are intact and likely to survive to ovulation.

**Conclusions:**

Combined, these improved, non-arbitrary methods will greatly improve our ability to estimate the fractions of growing/intact and non-growing/atretic follicles in mouse ovaries.

## Background

Ovarian follicles are the functional units of the organ [1–3], each containing an oocyte and associated somatic cells. Adjacent-most to the oocyte are the granulosa cells (GC) that grow in number during follicle development. Determining the number of intact (‘healthy’) follicles in an ovary at any time is a central measurement in reproductive biology. The number of intact follicles indicates the current reproductive status of the ovary, its relative ovarian ‘age’, and the impact of genetic or environmental modifiers upon follicle survival. Subtracting the number of follicles in an ovary that are undergoing death (termed atresia, ‘atretic’ follicles) from the total number reveals the number of intact follicles. Mammalian model organisms like the mouse, rat, and non-human primates have all been used as surrogates for relatively rare and precious human ovarian tissue.

Rodents are perhaps the most widely used model(s) for the study of the function of the mammalian ovary [3–6]. Mouse ovaries are easy to isolate and handle, and are amenable to the preparation of pole-to-pole serial histological sections that can be used to evaluate every constituent follicle. Because female mice exhibit reproductive cyclicity for just over a year, studies that span the entire reproductive lifespan are tractable. Upon reaching adulthood, the ovaries of common mouse strains contain on the order of 3000 to 5000 follicles of varying stages of development [7], [Uslu and Johnson, unpublished]. That number declines over time as most follicles die *via* atresia, with a smaller number surviving to ovulate each estrus cycle. Genetically-manipulated mouse strains have been invaluable in the search for genes and genetic elements that control follicle development and broader ovarian function [8, 9]. Quantitative effects of genetic or chemical/environmental factors can be evaluated by counting the follicles in serial histological sections. Such serial section follicle counting is often referred to as ‘histomorphometric’ analysis.

Evaluation of multiple replicate ovaries in blinded treatment and control group(s) is required to fulfill the need for statistical rigor in histomorphometric studies. Because of the time required–and the need for experienced, consistent personnel to perform the studies–attempts have been made to make counting follicles easier and faster. Today, the most commonly used protocols include regular sub-sampling of all available tissue sections and the use of a correction factor to estimate the number of follicles present in all sections. This approach has been used to quantify the number of ovarian follicles at different developmental stages [10, 11], between different mouse strains or in the presence of a gene knockout versus its same-strain wild-type (WT) controls, or, to detect differences after environmental/chemical/pharmacological insult [5, 12].

**Fig. 1:**
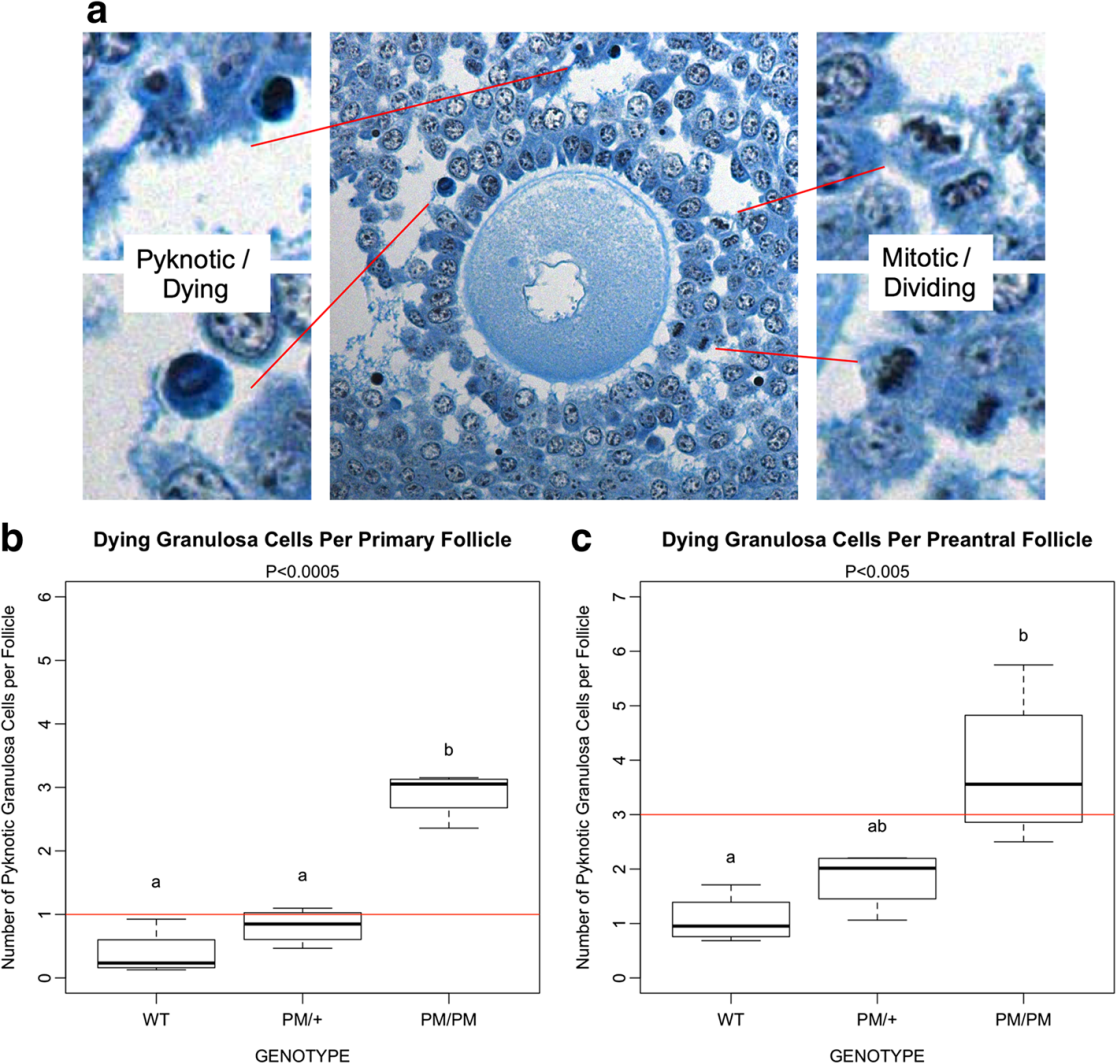
Follicles of homozygous Fragile X premutation mice contain elevated numbers of pyknotic nuclei. PM/PM ovarian follicles were found to contain more pyknotic nuclei than those from WT controls and PM/+ animals. Examples of mitotic (left insets at higher magnification) and pyknotic (right insets) granulosa cells are shown in the photomicrograph(s) in panel (**a**). PM/PM primary **b** and small preantral **c** follicles contained median numbers of pyknotic cells that exceed the ‘historical’ criteria for atretic follicles (*red lines*)

In 1978, Hirshfield and Midgley [13] established a method to determine the numbers of intact follicles in the adult rat ovary. In that study, atretic follicles in all developmental stages were identified–and excluded from the number of intact follicles–by the following cutoff criteria: “[The] follicle was considered to be undergoing atresia whenever 2 or more pyknotic GC could be found in a single section or whenever the oocyte showed obvious signs of degeneration, such as fragmentation, loss of the nuclear membrane or thinning of the cumulus oophorus.” This general approach was then extended to the mouse and slightly modified to account for increasing numbers of GC in the cross sections of small versus large follicles. The updated criteria [14, 15] held that “In small primary follicles, if one pyknotic GC nucleus was found in the largest cross section and in large antral follicles, if three pyknotic GC nuclei were seen, the follicle was scored as atretic [and was excluded from the count of intact ‘healthy’ follicles]” (Fig. 1). This strategy where a cutoff number [16] (or more recently, a percentage [17]) of dying cells is the determinant of follicle survival has been the standard since the original 1978 paper; we refer to these as the “Historical criteria.” Advances in labeling biochemical markers of cell death like active Caspase 3 [18, 19], or, degraded genomic DNA using the TUNEL assay [20, 21] offer confirmation of GC death; however, these methods have not added significant information beyond the histological evaluation of pyknotic nuclei in terms of our ability to identify which follicles have committed to atresia.

In a recent revisiting of histomorphometric follicle analysis, Myers et al. [22] tested a ‘fractionator/physical’ design. This method was applied to the ovaries of 70-day-old mice to estimate primordial and primary follicle number and also to count zona pellucida (ZP) remnants (ZPR) that remained after the atresia of mature follicles. The ZP is an extracellular matrix layer produced specifically by the oocytes of growing follicles, and primarily consists of an assembly of three glycoproteins, ZP1, ZP2, and ZP3 [see [23] for a review]. Every 10th and 11th section were chosen from a random start, and analysis of the sections was bidirectional so that both were ultimately used as look up and reference sections. Quantification of secondary and antral follicles was done using exact counts of consecutive sections, and the presence of an oocyte nucleus was used as a proxy for and intact follicle. The number of atretic follicles per ovary was estimated by using the fractionator/physical design by counting ZPR.

Estimation of the numbers of ZPR is at least as reliable (if not more so due to their obvious appearance) as estimating the total number of follicles. However, the number of follicles that had more recently committed to atresia is unaccounted-for. What of the follicles whose GC or oocytes have only recently begun to degenerate? Stated another way, ZPR represent the end stages of the atresia of quite mature follicles that contained an oocyte with an intact ZP matrix. This is because immature follicles of the primordial, primary, and early secondary stages contain oocytes that lack mature ZP and thus do not leave a ZPR behind after their death. RNA [24] and protein [25] localization studies have shown that ZP are not present around the oocytes of murine primordial follicles. While ZP2 mRNA has been detected in primordial follicle oocytes, ZP1 and ZP3 expression begins when follicle growth activation occurs. Subsequent secretion, development of ZP protein filaments, and patchy assembly of ZP matrix follows during early follicle growth [26].

Development of an intact, oocyte-surrounding ZP likely occurs early in follicle development, but exactly when a ZP arises that remains as a ZPR after follicle atresia is unclear. If a follicle undergoes atresia prior to this time, no ZPR will remain as evidence of its demise. Paraphrasing Osman’s 1985 [27] mention of these challenges is instructive: “Recording all atretic follicles, including those in advanced stages of atresia, does not provide information about the time of onset of atresia since considerable time could have elapsed before the affected follicles showed signs of the advanced stages of atresia such as pseudo-maturation spindle or fragmentation of the oocyte [or development of a ZPR].”

In our hands, a recent evaluation of a strain of mice that harbor Fragile X Premutation (FX PM)-length CGG repeats [28] revealed significant mitochondrial abnormalities and reduced mitochondrial content in the GC and oocytes of mutants that were lacking in same-strain WT controls [29]. In homozygous mutants (PM/PM), almost all primary ovarian follicles contained greater than or equal to one pyknotic nucleus and greater than or equal to three pyknotic nuclei in larger follicles. As this genotype of the mouse strain had approximately normal follicle numbers *at all growth stages* throughout postnatal life compared to WT same-strain controls, it was not possible that all follicles that were identified as atretic were actually dying. Instead, it seemed that even growing/intact FXPM follicles contained a preponderance of pyknotic GC, that correlated with their mutant genotype. To attempt to resolve this, we performed a more careful examination of the disposition of all GC in representative follicles and ZPR in the ovaries of FXPM mice and controls.

By taking the number of mitotic GC into account, we have developed an improved method of identifying ovarian follicles that are growing versus those that are not growing, and in some cases that have believably committed to atresia. Comparison of pyknotic and mitotic GC numbers in antral follicles led to the realization that two distinct populations of antral follicles are present, differentiated by their shape in the largest cross-section. The shape of antral follicles was found to strongly correlate with the number of dying GC. Overall, we show that this method reduces false-positive and false-negative mis-classification of growing/intact and non-growing/atretic follicles, while not being more labor- or time-intensive than current approaches. In this way, we and others can improve our estimation of ovarian follicle population(s) in the contexts of aging, genetic manipulation, or insult.

## Methods

### Statement of ethics, animals, and preparation of histological specimens

C57Bl/6 and FX130R (PM) mice were handled under an approved animal protocol at the Yale School of Medicine (#2013-11569). A minimum of 4 females per genotype (WT, PM/+, PM/PM) were produced. At 8 months of age, ovaries were collected for processing after [7]. Briefly, ovaries were fixed in Dietrich’s Fixative for a minimum of 12 hrs at 4 °C, then stored in 70% ethanol prior to further dehydration in an ethanol series, followed by Xylenes and embedding in paraffin blocks. Serial 5 micron sections were cut using a microtome, and were prepared in order onto positively-charged glass slides. Sections were melted for 10 mins at 55°, and then rehydrated and stained with Weigert’s Iron Hematoxylin and Methyl blue, followed by a final dehydration and mounting using Permount.

### Methodology for identifying growing versus non-growing ovarian follicles

**Fig. 2:**
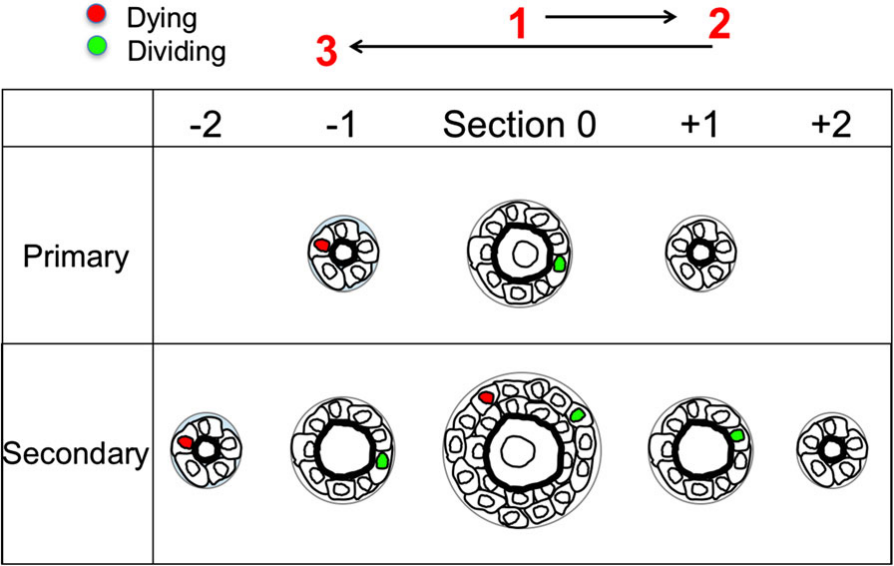
Cartoon scheme of preliminary follicle evaluation and counting method. To determine the total number of mitotic and pyknotic figures in follicle GC, the central section (e.g., the section containing the oocyte’s nucleus) was identified and serial sections were first evaluated in one direction, and then the other. Mitotic and pyknotic figures were scored for each follicle in this fashion

For the development of the method, all follicles were evaluated in each ovary as follows (summarized in cartoon in Fig. 2). First, the largest/center section of each follicle was identified. If the oocyte showed signs of degeneration or fragmentation, the follicle was scored atretic, and mitotic and pyknotic nuclei were counted in all serial sections. If the oocyte was intact, all pyknotic nuclei in the largest section were recorded, as well as all mitotic figures. Each additional serial section of each follicle was evaluated, and again all pyknotic and mitotic figures were recorded. This was done using a customized “counting sheet” that designated the largest/center section as position 0, and adjacent sections in one direction in “minus” positions and adjacent sections in the opposite direction “plus” positions. For example, a small primordial follicle might consist only of three serial sections designated –1, 0 (center), and +1, while a larger small antral follicle might have 8 sections designated –4, –3, –2, –1, 0, 1, 2, 3. Evaluation of the complete dataset of pyknotic and mitotic figures allowed the retrospective testing of criteria for designating follicles growing/intact or non-growing/atretic, and the determination of the accuracy of said criteria for false positive and negative rate(s).

### Data processing and statistical analyses

After collection, data were entered into spreadsheets and were saved as raw comma-delimited files. R [30] was used for data processing, the production of charts, and for all statistical analyses. Raw project data as well as scripts used for data analysis are available upon request.

## Results

### Analysis of pyknotic figures in the ovarian follicles of a mouse model of Fragile X Premutation

Recently, one of our laboratories performed a confirmatory histomorphometric analysis of follicle numbers in a mouse model of Fragile X Primary Ovarian Insufficiency (FXPOI/FX130R mice) [28]. Prior analysis tested the hypothesis that the ovaries of animals heterozygous for FXPM would have a lower primordial ‘reserve’ of follicles in FXPM mice compared to WT controls, as is postulated to occur in the human FXPOI condition. Instead, that study found only a slight (but significant) decline in the number of primordial follicles between 7 and 9 months of age that returned to WT numbers between 12 and 14 months of age. Wondering whether animals homozygous for the FXPM would have a more significant impact on follicle numbers, we performed a blinded analysis on 8-month-old WT, heterozygous PM (PM/+) and homozygous PM (PM/PM) animals [29]. As in prior studies, we used the ‘historical’ criteria to identify intact and atretic follicles.

During blinded counting, we noticed that several serial section preparations (*n*=4 ovaries per genotype) contained immature (primordial, primary, small preantral) follicles that, if evaluated using ‘historical’ criteria considering pyknotic granulosa nuclei would almost all be categorized as atretic. Examples of pyknotic GC in a histological preparation prepared as mentioned in Methods (above) are shown in Fig. 1 a, left insets. Upon decoding the slides, we found that all of these were PM/PM ovaries. Considered as a percentage, PM/PM ovaries contained approximately 90% primary follicles with greater than or equal to 1 pyknotic nucleus in the largest cross section and also 80% preantral follicles with 3 or greater pyknotic nuclei. Boxplots are shown in Fig. 1 that summarize the median numbers (and quartiles) of pyknotic nuclei of WT, PM/+, and PM/PM primary follicles (1B) and small preantral follicles (1C). Mean numbers (± standard error, S.E.M.) of PM/PM pyknotic nuclei in primary and small preantral follicles were 2.90 ±0.18 and 3.84 ±0.70, respectively.

We initially interpreted this result as evidence that homozygosity for the FXPM corresponds to increased atresia of immature follicles. However, when all immature follicles, regardless of pyknotic nuclei content, were considered, PM/PM ovaries contained similar numbers at each stage through antrum development [28]. Even if follicle atresia is recognized at its earliest using these historical cutoffs for pyknotic nuclei, the number of follicles must be reduced in successive stages if the death process has been activated. That is, if an immature follicle commits to atresia, it cannot also survive and grow to be present at successive stage(s). We therefore posited that instead of a situation where almost all immature follicles of the PM/PM mice had simultaneously committed to atresia at 8 months of age, that elevated numbers of dying GC are present in *surviving/growing* PM/PM follicles compared to WT follicles. The next important question, then, was whether we could determine which PM/PM follicles had actually committed to atresia, and which were instead intact *and growing* despite an increase in GC death relative to WT controls.

We reasoned that consideration of both the number of pyknotic nuclei and the number of mitotic cells (examples of GC mitotic figures are shown in Fig. 1 a, right insets) might reveal at least whether the GC population was growing in number in each follicle, despite the number of dying cells counted. If the oocyte is intact and more GC mitotic figures were present than pyknotic nuclei, that follicle’s GC population was definitively growing in number, and we could justify categorizing that follicle as growing/intact and not atretic. Conversely, if fewer mitotic figures were present than pyknotic nuclei, we could consider that follicle at least non-growing and possibly atretic. Summarized results from our preliminary studies and our development of a new approach for evaluating follicle status follows here.

### Pyknotic nuclei and mitotic figures in primary, secondary, and small preantral follicles

Our evaluation of dying and dividing cells within immature follicles was planned as follows. Initially, we evaluated each serial section for each ovary (*n*=3 per genotype). In each section, we evaluated each primordial, primary, secondary, and small preantral follicle. We designated the center section that contained the oocyte’s nucleus as section “zero/0.” If the oocyte was fragmented, we could immediately score the follicle as atretic. The most adjacent serial sections were –1 in one direction and +1 in the opposite direction; surrounding serial sections were thus –1, –2, –3, etc. in one direction, and +1, +2, +3, etc. in the other direction. For each section, we recorded the number of pyknotic and mitotic figures. Ovary fixation in Dietrich’s fixative, and staining of tissue sections using Weigert’s Iron Hematoxylin chromatin staining and Methyl blue counterstaining per our standard protocol results in clear and easy-to-identify pyknotic and mitotic figures with minimal training and some practice. Standard 4% paraformaldehyde fixation and hematoxylin/eosin counterstaining does not result in the same quality of subcellular detail and thus we do not use or recommend it.

This scheme allowed a total catalog of all cells of either disposition in each section, and also allowed us to compare these data with the “historical” method that only takes the presence or absence of pyknotic nuclei in the center section “0” into account. A cartoon summary of this strategy for immature follicles is shown in Fig. 2. Again, if the number of mitotic GC in the entire follicle was greater than or equal to the number of pyknotic nuclei, we could infer that that the total GC population was increasing, and thus the follicle was growing. If instead the number of pyknotic nuclei exceeded the number of mitotic figures in a follicle, the GC population was declining, and it was scored as non-growing/atretic.

**Fig. 3:**
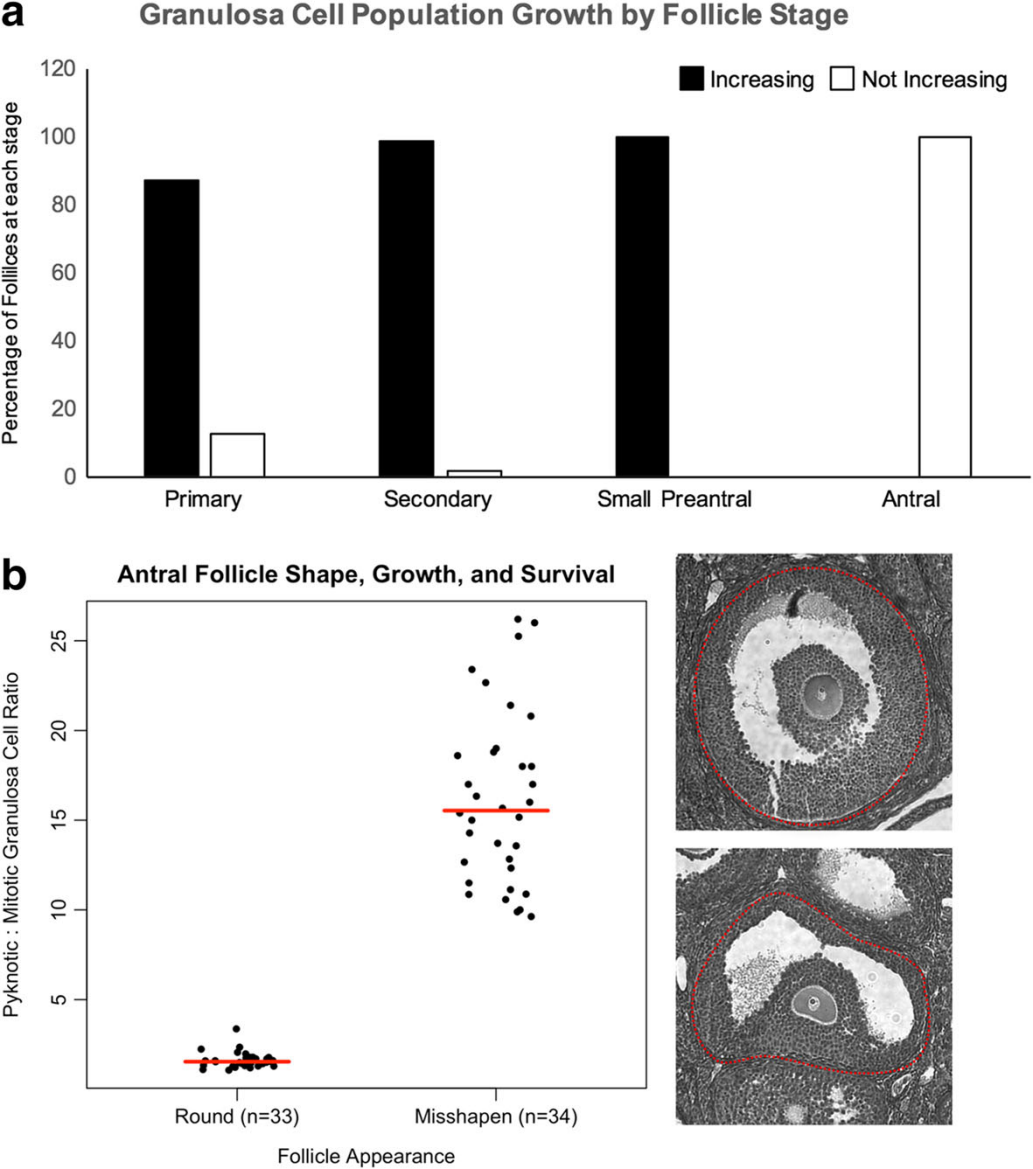
Identification of Growing/Intact and Non-Growing/Atretic follicles. (**a**) Using the scheme summarized in Fig. 2, follicles in each developmental category in (*n*=3 ovaries) were evaluated in terms of the ratio of pyknotic to mitotic figures, and expressed as GC population “Increasing” (*black bars*) or “Not Increasing” (*white bars*). Panel **b** shows the ratio of pyknotic to mitotic GC in antral follicles after dividing into those that were subjectively “round” by eye (*n*=34, example in *upper right photomicrograph*) and those that were misshapen (*n*=35, example in *lower right photomicrograph*) in the center section containing the oocyte nucleus. Median ratios are indicated with *red lines*. Ratios were found to be significantly different between round and misshapen antral follicles by Student’s t-test, P <2.2e ^−16^

It was apparent that the numbers of pyknotic and mitotic figures per immature follicle were bimodal. In almost every case, follicles either had a slight deficit of pyknotic figures relative to mitotic figures, or, had a large excess of pyknotic figures relative to mitotic figures. When expressed as the ratio of pyknotic GC to mitotic GC according to follicle growth stage (Fig. 3 a) we could see that for some primary and secondary follicles, the GC population was not increasing (non-growing follicles), and that all small preantral follicles had more mitotic figures than pyknotic figures and therefore contained GC populations that were increasing (follicles that were considered growing).

While the determination of pyknotic and mitotic figures in immature follicles was informative, we found that analysis of the greater number of serial sections of antral follicles was both more time consuming and characterized by a different pattern of GC death and division. After antrum development, the total number of follicle GC pyknotic figures always exceeded the number of mitotic figures (Fig. 3 a, ‘Antral’). Antral follicles are well-known to contain many pyknotic GC adjacent to the antral space. Because some fraction of antral follicles will definitively survive to ovulate, two interesting points are raised. First, the pyknotic:mitotic GC ratio cannot be used to identify non-growing follicles in the same way as used for immature follicle stages. Second, a lack of GC population growth during antrum formation indicates that the acquisition of greater volume over time that antral follicles exhibit can be attributed to greater antral fluid content, and not to increased cellularity. It remained to be seen then, whether we could identify non-growing/atretic antral follicles.

### Mitotic and pyknotic figures in each serial section of antral follicles: the shape of antral follicles correlates with their GC population growth status

Dying and dividing GC numbers again appeared bimodal in antral follicles, with some follicles containing approximately equal numbers and some that contained many more pyknotic than mitotic figures. After careful evaluation of different antral follicles, we noted that the cross-sectional shape or ‘round-ness’ of antral follicles strongly correlated with their GC content in terms of death and proliferation.

Follicles with a subjective round appearance in the largest cross section had high numbers of mitotic figures (52.77 ±0.49; mean ±S.E.M., *n*=34 follicles) and also a relatively low ratio of pyknotic : mitotic figures per follicle (1.64 ±0.07; Fig. 3 b, example “round” follicle in upper adjacent panel). Follicles that were very obviously not round, and instead had an oblong or deformed shape in their largest cross-section had significantly fewer mitotic figures per follicle (6.31 ±0.05) and a much higher ratio of pyknotic : mitotic figures per follicle (16.06 ±0.81; Fig. 3 b, example in lower adjacent panel). Interestingly, while oocyte fragmentation is relatively rare in antral follicles overall, the few examples of fragmenting oocytes that we did note in this set of 67 follicles evaluated pole-to-pole were all (7/7) found in misshapen antral follicles. This finding was consistent with the idea that antral follicle shape is a simple indicator of follicle status where that round follicles are nearly always growing/intact and misshapen follicles are much more likely to be non-growing/atretic.

### Empty follicles

**Fig. 4:**
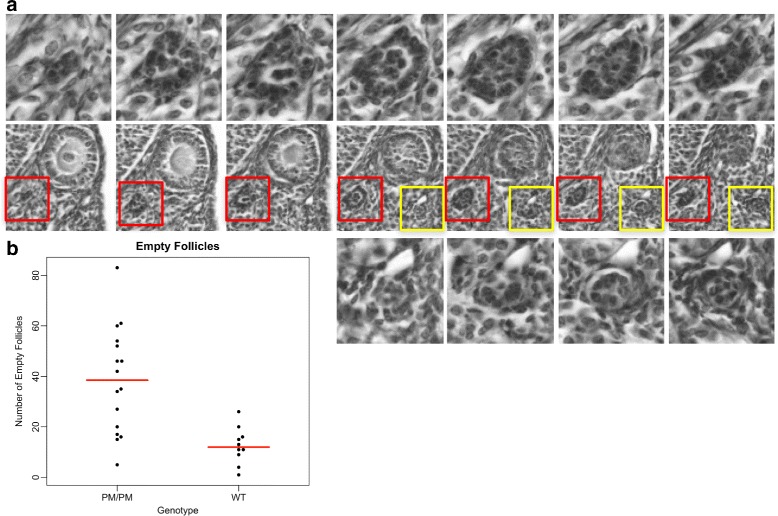
Quantification of empty follicles during serial section analysis of WT and PM/PM ovaries. Follicle structures were identified that consisted of granulosa cells but did not show any sign of an oocyte in any serial section. An example of two empty follicles in a WT sample is shown in the serial sections in panel (**a**). The center row of photomicrographs is at lower magnification with matching serial images of a single empty follicle above (*red boxes*) and another below (*yellow boxes*) at higher magnification. Panel **b** is a dotplot of total numbers of empty follicles counted in individual blinded specimens in WT and PM/PM ovaries; median empty follicle numbers are indicated by *red lines*

During our serial section analysis we noted that some specimens contained structures that were composed of granulosa cells but that either did not contain any sign of an oocyte or only contained a *zona pellucida* remnant. We referred to those structures as empty follicles and included their quantification in our analysis. Representative images of empty follicles in a WT ovary are shown in Fig. 4 a, insets at higher magnification are shown above and below the center row at lower magnification. After de-coding our WT and PM/PM specimens, we found that PM/PM (*n*=16) ovaries contained significantly more empty follicles than WT controls (*n*=10) (mean ±S.E.M., 38.31 ±5.26 versus 12.60 ±2.30, respectively, significantly different by Student’s t-test, P <2.3e ^−4^). These data are summarized in Fig. 4 b, a dot plot where median numbers of empty follicles are depicted by red lines.

### Development of follicle counting scheme that accounts for GC proliferation/follicle growth

**Fig. 5:**
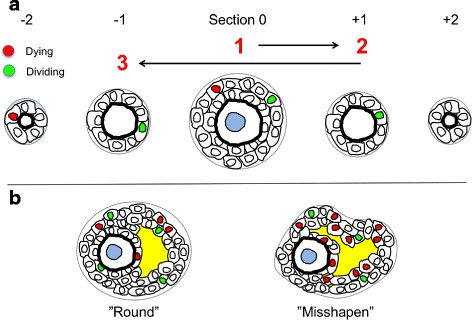
Development of follicle counting scheme that accounts for GC proliferation/follicle growth. **a** For preantral follicles: i. If the oocyte is fragmenting or degenerate, score the follicle Non-growing/Atretic. 1. Count all GC mitotic figures in the center section that contains the oocyte nucleus (*light blue*). If >= 1 dividing GC, the follicle is scored Growing/Intact. Go to step 2. 2. If zero dividing GC in center section, count mitotic and pyknotic GC in sections 0, +1, then -1. If a total of 2 dividing GC is reached, the follicle is Growing/Intact. Go to step 3. 3. The follicle is Non-growing/Atretic if fewer than 2 dividing GC are found, or, dying cells >= dividing cells. **b** For antral follicles: For antral follicles, find the center section that contains the oocyte nucleus. If the oocyte is fragmenting or degenerate, score the follicle Non-growing/Atretic. Score round follicles Intact, and misshapen follicles Atretic

We took all of the above information into account and developed a follicle counting protocol, evaluating follicles every fifth serial section. This protocol is summarized in cartoon form in Fig. 5.

**Immature (pre-antral follicles:)**

1. Find each follicle’s center section that contains the oocyte’s nucleus. If the oocyte is of degenerate appearance, or is fragmenting, score the follicle Non-growing/Atretic. If the oocyte is intact, go to step 2.

2. Count all dividing GC in the center section. If >= 1 dividing cell, the follicle is scored Growing/Intact. If zero dividing cells in center section, go to step 3.

3. Evaluate sections –1 and +1 relative to the center section. No non-growing follicle has been identified with > =2 dividing cells in the three center-most serial sections. Thus if a total of 2 dividing cells is reached, the follicle is Growing/Intact. If 2 dividing cells are not identified, count dividing and dying cells in remaining serial sections if applicable and go to step 4.

4. After all sections have been evaluated, compare the numbers of pyknotic and mitotic figures. The follicle is Non-growing/Atretic if dying cells >= dividing cells, and the follicle is Growing/Non-Atretic if dying cells < dividing cells.

**Antral follicles:**

5. For antral follicles, evaluate their shape in the central-most serial section that includes the oocyte nucleus. A follicle that is roughly round in appearance with an intact, non-fragmenting oocyte is scored growing/intact. A follicle that either contains a fragmenting oocyte, or, is misshapen (e.g., is not subjectively round) is scored non-growing/atretic. Examples of each antral follicle type are shown in Fig. 3 b.

**Empty follicles:**

6 (optional). Empty follicle quantification is optional. Here, GC structures for which an oocyte cannot be found, or, only a *zona pellucida* remnant is present during serial section evaluation are scored as empty follicles. Because of their relative scarcity at approximately 10 per ovary in WT C57Bl/6 specimens, empty follicle contribution to follicle loss over time can be considered minimal. However, our finding that they are approximately four-fold more common in PM/PM specimens suggests that the development of empty follicles may be different under different conditions. As these structures would evade detection using the Historical method, any contribution to overall follicle loss would be missed without such careful serial evaluation. We suggest that empty follicles be evaluated on a case-by case basis.

### Comparison between follicle counting methods

As mentioned, identification of mitotic and pyknotic bodies in tissue sections prepared in this fashion is highly accurate due to their unambiguous appearance(s). With practice, we found that the speed that follicle content within ovaries could be assessed was comparable between the two methods as well. This was because only a subset of counted follicles needed to be evaluated beyond the center section. What remained was a head-to-head comparison between the counting methods. We needed to determine how similar or different each method was in the quantification of growing/intact follicles in entire ovaries, and how accurate each method was for individual follicles.

**Fig. 6:**
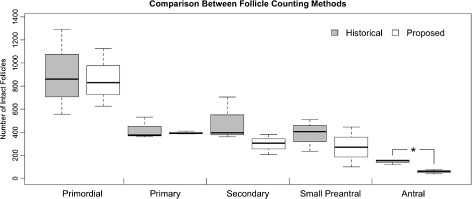
Comparison of methods for quantification of immature follicles in PM/PM ovaries and WT controls. Total numbers of primordial, primary, secondary, small preantral, and antral follicles are shown as determined by the ‘Historical’ method (*grey bars*) and the method ‘Proposed’ here (*white bars*). No significant difference was seen in primordial, primary, secondary and small preantral follicle types between counting methods. The Proposed method resulted in significantly fewer antral follicles designated Intact than the Historical method (P <0.05, Student’s t-test)

We counted primordial, primary, secondary, small preantral, and antral follicles in the ovaries of 8 month in every 5th section as described. A boxplot is shown in Fig. 6 that summarizes median follicle numbers (dark central line in boxes) and quartiles (box upper and lower boundaries, whiskers) of follicles at each stage when the Historical (grey boxes) and Proposed methods were used. Follicle numbers were similar in all categories except for antral follicles, which were significantly reduced in number when the Proposed method was used compared to the Historical method. We attribute this to the inclusion of antral follicles that both were ‘misshapen’ and contained the higher numbers of pyknotic GC relative to mitotic GC (Fig. 3 b) in the intact category due to the Historical method’s ignoring these features.

To compare the *accuracy* of follicle categorization between the methods, we needed to quantify the rates at which follicles were mis-identified. For example, how many follicles are incorrectly categorized “non-growing/atretic” using the historical method (false positives) that are actually growing? How many follicles are incorrectly categorized “growing/intact” that actually have more pyknotic figures than mitotic? Because our data recording sheets include the number of pyknotic and mitotic figures in each section, we could determine how often the lack of a one or three dying GC in the center section

To our surprise, the historical criteria performed very poorly, with data summarized as follows. A single pyknotic nucleus in the center section corresponded to an approximate 50% false positive rate where mitotic figures showed that the follicle was instead growing. When primary follicles were considered alone, 9 of 81 primary follicles were scored non-growing/atretic due to 1 or more dying GC per largest cross-section. However, 8 of those 9 follicles were found to be “false-positives” that we could confirm were instead growing. There were an average of 3.5 dividing cells per follicle and 1.1 dying cell per follicle (one always in largest cross section). Further, the historical criteria did not identify 6.3 follicles per ovary that were not growing (false negatives). Although these follicles did not have a dying cell in the largest cross section, our method showed that indeed the number of dying cells was greater than the number of dividing cells. In the end, we were surprised that the Historical method resulted in a reasonable estimation of follicle number of all classes except for those at the antral stage, given its poor accuracy in categorizing individual follicles.

## Discussion

If the population of follicles that are present in an ovary at a given time is to be assessed, the non-growing/atretic fraction must be determined accurately. As has been shown here, the use of cutoff values for pyknotic GC nuclei–when the oocyte is intact in a follicle–results in an unacceptably high rate of false positives called atretic, and false negatives incorrectly called intact in WT ovaries. This is because as long as mitotic figures exceed pyknotic figures, it is not possible that the GC population is declining at that time. Instead, that follicle’s GC population is growing. Evaluation of the FXPM double mutant further shows that a threshold approach for calling follicles atretic can have striking exceptions. It is not possible that all immature follicles of 8-month-old PM/PM mice have simultaneously committed to atresia as the mice exhibit normal ovarian function and an approximately normal fertile lifespan compared to WT controls. Our method, that evaluates mitotic figures in at most, three serial sections of immature follicles, accurately identifies those follicles that are growing, and also those that are not.

A remarkable feature of these data is that despite the poor accuracy of identifying individual mouse follicles as growing/intact or non-growing/atretic, the Historical method performs quite well when an estimate of follicle number is needed. Choosing thresholds for dying GC allows a reasonable estimation at least for WT ovaries as the lack of significant differences between the methods (Fig. 6) for all but antral follicles makes clear. However, if the impact of genetic or environmental factors upon follicle development is to be understood beyond a superficial level, one cannot rely on threshold numbers of dying GC. Our own data on FX PM follicle numbers (Fig. 1) shows that increased GC death does not necessarily mean increased follicle atresia.

The more precise evaluation of FXPM double mutants allowed by our proposed method has led us to modify our interpretation of the phenotype. The detection of increased numbers of pyknotic GC in growing follicles in the presence of the PM suggests that that cell viability is compromised by mitochondrial abnormalities in cells that express PM RNA [29]. While minimal impact upon follicle number was detected, this follow-up study shows that GC survival within follicles is indeed compromised, and confirms an earlier observation that cumulus GC number is decreased in PM ovaries [28]. Could such a defect in GC survival impact oocyte development and/or quality?

The major pitfall of our method can be found in the hypothetical situation where an immature ovarian follicle might temporarily halt all GC division, only for proliferation to resume at a later time. This follicle would almost certainly contain dying GC, as no follicle beyond the primordial stage that has **neither** dying nor dividing cells has been noted in our studies to date. GC proliferation in immature follicles exhibits the same 18–24 hr cell cycle (as extrapolated from mitotic index) [31] as found in most other cell types [32]. If a follicle has more than 18–24 cells, and the visible stages of M-phase last approximately one hour, we can expect at least one mitotic figure in a non-synchronized population. We suspect that if follicles can halt growth and then resume it later, that this will be a relatively rare occurrence that will not significantly impact overall quantification and interpretations.

Our interpretation that follicles that entirely lack mitotic GC have committed to atresia is also supported by the status of follicles that contain degenerate oocytes. We have only very rarely identified follicles that contain a degenerate oocyte that also contain even a single mitotic GC. In these cases where the commitment to atresia is clear, it is even less likely that GC proliferation could re-start after halting. Despite granting that there may be exceptions to this where all of the GC of an intact follicle could halt proliferation only to resume later, we consider halted proliferation in the presence of only pyknotic nuclei consistent with a commitment to atresia.

It should be emphasized that our initial finding that for eight-month-old C57Bl/6 animals, numbers of follicles were found to be similar whether the Historical method or our proposed method was used. Indeed, only antral follicles differed significantly, and their reduction when the proposed counting method was used can be directly attributed to mis-classification of those antral follicles with the highest number of dying GC as growing/intact. It was striking that the ‘round-ness’ of antral follicles was such an effective determinant of granulosa cell survival (Fig. 3 b). Intuitively, evenly distributed GC death relative to proliferation should correspond to a more ‘round’ follicle. This finding is worthy of further exploration in terms of overall follicle health, and perhaps, oocyte quality after retrieval from antral follicles. One might predict that the ‘rounder’ the follicle, the more likely that an oocyte would also be intact and of higher quality. It remains to be seen whether these data can be extended to the human context of assisted reproduction.

Using modern tools we are in position to greatly improve our understanding of factors that control follicle ‘dynamics’ during the mammalian reproductive lifespan. To accomplish this, rapid and accurate evaluation of the fraction of follicles in the ovary that is undergoing atresia is needed. While our technique cannot guarantee total accuracy when non-growing follicles that contain intact oocytes are present, it is a large step forward compared to apparently arbitrary cutoffs for numbers of dying GC. Perhaps one day a non-invasive method will be developed that can accurately count numbers of intact and atretic follicles of all developmental stages in live animals, and also women. Until that time, histomorphometric analyses will remain necessary, powerful tools.

## Conclusions

A detailed evaluation of all pyknotic and mitotic figures in WT ovarian follicles and those from FX130R mice showed that identification of at least one mitotic figure in the largest cross section, and at most, one additional adjacent section, accurately identifies those (non-primordial) follicles that are growing. In the absence of mitotic figures, pyknotic nuclei are likely to indicate that the follicle has committed irreversibly to atresia. Subjective evaluation of “round” follicles to be considered growing/intact and “misshapen” follicles to be considered non-growing/atretic is a rapid and accurate way to quantify antral follicles. A trained investigator using a relatively inexpensive sample preparation protocol and a simple counting worksheet can rapidly and accurately quantify both the total number of follicles of different developmental stages, and also the fractions of follicles that are intact or atretic. While evaluation using the ‘historical’ criteria can be used to generate a reasonable estimate of total and dying follicles, the example of the FXPM animal shows that exceptions where significant mis-identification of follicle life and death can take place. Application of this strategy to human ovarian specimens of sufficient histological quality should lead to more accurate determination(s) of the impact of genetic or environmental factors upon follicle growth and survival in women.

## Abbreviations

FX: Fragile X
FXPM: FX premutation
FXPOI: FX primary ovarian insufficiency
FX130R: FX 130-repeat harboring mice
GC: Granulosa cells
WT: Wild-type
ZP: Zona pellucida
ZPR: ZP remnants
